# Association of *MTHFR* polymorphism, folic acid, and vitamin B12 with serum homocysteine levels in pregnant women

**DOI:** 10.17305/bb.2023.9260

**Published:** 2024-02-01

**Authors:** Ana Bošković, Ana Ćuk, Vedrana Mandrapa, Ana Dugandžić Šimić, Ivona Cvetković, Martina Orlović Vlaho, Tanja Krešić, Tanja Tomić, Vajdana Tomić

**Affiliations:** 1 Department of Obstetrics and Gynecology, University Clinical Hospital Mostar, Mostar, Bosnia and Herzegovina; 2 Department of Laboratory Diagnostics, University Clinical Hospital Mostar, Mostar, Bosnia and Herzegovina; 3 Faculty of Health Studies, University Mostar, Mostar, Bosnia and Herzegovina; 4 Faculty of Medicine, University Mostar, Mostar, Bosnia and Herzegovina

**Keywords:** Methylenetetrahydrofolate reductase (*MTHFR*) polymorphism, folic acid, vitamin B12, homocisteine, pregnancy

## Abstract

Homocysteine is known to be associated with adverse vascular and metabolic effects, as well as pregnancy complications. Its serum levels are influenced by the function of the enzyme methylenetetrahydrofolate reductase (MTHFR) and the dietary intake of folic acid, vitamin B12, and methionine. In this cross-sectional study, we investigated the association of genetic polymorphisms of the *MTHFR* gene with vitamin status in pregnant women during mandatory folic acid supplementation. The study included 102 pregnant women between 24 and 28 weeks of gestation who were attending regular outpatient examinations at the maternity clinic. Homocysteine, folic acid, vitamin B12 levels, and *MTHFR* gene polymorphisms (C677T and A1298C) were analyzed. Significant associations were found between vitamin B12 and folic acid levels with homocysteine (*P*< 0.001), with lower serum levels of these vitamins being associated with higher homocysteine levels. Surprisingly, there was no significant association between *MTHFR* genetic polymorphisms and serum homocysteine levels, likely attributed to the supplementation of folic acid and vitamin B12 in vitamin supplements for pregnant women, which counteracts the effect of the mutation. Remarkably, a high prevalence of *MTHFR* gene mutations was found, with the C677T polymorphism present in 56.9% and the A1298C polymorphism in 87.2% of pregnant women. These findings emphasize the importance of adequate folic acid and vitamin B12 intake during pregnancy to regulate homocysteine levels. Although the *MTHFR* gene mutations were highly prevalent, their influence on homocysteine levels in this population appears to be mitigated by vitamin supplementation. Further research is warranted to explore the impact of these mutations on other aspects of pregnancy outcomes. The trial is registered at Clinicaltrail.gov (NCT04952324).

## Introduction

In recent decades, there has been a growing scientific interest in understanding the role of pathologic endothelial activation in disease development [1]. Among the markers of vascular dysfunction, homocysteine has emerged as a significant factor [2]. Elevated levels of homocysteine are associated with numerous adverse vascular and metabolic effects [3–7]. Homocysteine is produced during the metabolism of methionine, an essential amino acid obtained from the daily diet. Several factors influence its levels, including diet, age, smoking, renal dysfunction, and hypothyroidism. However, one of the leading causes of hyperhomocysteinemia is the genetic polymorphism in the methylenetetrahydrofolate reductase (*MTHFR*) gene [8].

MTHFR plays a crucial role in the methylation cycle and is involved in the biochemical pathways of methionine and folate. It catalyzes the conversion of 5,10-methylenetetrahydrofolate into 5-methyltetrahydrofolate, which is necessary for the remethylation of homocysteine to methionine [9]. Dysfunctional MTHFR activity can have wide-ranging effects, impacting DNA and amino acid synthesis, DNA methylation, gene expression, and cellular function [10]. These disruptions may lead to increased gene expression of inflammatory molecules, post-translational modifications of proteins, and the induction of mitochondrial dysfunction, potentially contributing to various diseases [11, 12].

Within the *MTHFR* gene, certain common polymorphisms, such as the C677T and A1298C mutations, are prevalent in the general population. Homozygotes for *MTHFR* 677CT have only 10%–20% enzyme activity, and heterozygotes have 60% enzyme activity [13]. Reduced MTHFR activity, especially in the context of decreased folic acid and vitamin B12 intake, can result in hyperhomocysteinemia with its associated adverse effects. Notably, hyperhomocysteinemia has been linked to adverse pregnancy outcomes, including preeclampsia, fetal growth retardation, recurrent pregnancy loss, and placental abruption [14]. However, the impact of *MTHFR* gene polymorphism on homocysteine serum levels may be modulated by vitamin supplementation [15]. Vitamin B12 and folic acid intake have been shown to significantly reduce the influence of the mutation on homocysteine levels. As such, mandatory folic acid supplementation during pregnancy may play a crucial role in mitigating the adverse effects of MTHFR genetic variations [16].

The primary objective of this cross-sectional study is to investigate the association between *MTHFR* gene polymorphism and the levels of folate and vitamin B12 with the serum levels of homocysteine in pregnant women. Additionally, we aim to determine the frequency of the C677T and A1298C polymorphisms in the Herzegovinian population of pregnant women. Understanding these associations may shed light on the interplay between genetic factors, vitamin status, and homocysteine levels during pregnancy, potentially influencing maternal and fetal health outcomes.

## Materials and methods

### Study design and participants

A cross-sectional study was conducted to investigate the association between genetic polymorphisms of the *MTHFR* gene and serum levels of homocysteine, vitamin B12, and folic acid in pregnant women. The study population comprised 102 pregnant women aged between 19 and 43 years. Participants were randomly selected from those attending regular outpatient examinations at the maternity clinic between 24 and 28 weeks of gestation, with pre-pregnancy body mass index (BMI) ranging from 18.5 to 24.9 kg/m^2^. Exclusion criteria were smoking and multiple pregnancies.

### Sample collection and analysis 

Peripheral blood samples were collected from each participant during regular examinations at the Department of Gynecology and Obstetrics of the University Clinical Hospital Mostar. Blood was collected in two types of tubes: a tube without an anticoagulant (7.5 mL; serum) and a tube with ethylenediaminetetraacetic acid (EDTA) (2.6 mL; whole blood). The serum sample was obtained by centrifuging the blood at 3500 rpm for 10 min and then stored at −20 ^∘^C until analysis. The concentrations of folic acid, active B12 (holotranscobalamin), and homocysteine were measured from the serum samples using a chemiluminescent microparticle immunoassay (CMIA) on an Architect i4000SR analyzer (Abbott, Germany). Folic acid and active B12 levels were measured in nmol/L and pmol/L, respectively, while homocysteine levels were measured in µmol/L.

### Genetic analysis 

DNA extraction was performed from whole blood samples collected in EDTA tubes using the Purelink^TM^ Genomic DNA Mini Kit (Invitrogen, K182002, USA). The genomic DNA was eluted in 70 µL of distilled water and stored at −20 ^∘^C until analysis. DNA quantification was conducted using a Qubit 4 fluorometer with the Qubit dsDNA BR Assay KIT. The *MTHFR* gene polymorphisms (C677T and A1298C) were analyzed using Applied Biosystems^TM^ TaqMan^®^ SNP Genotyping Assays and polymerase chain reaction (PCR). TaqMan^®^ 5-nuclease was utilized to amplify and detect specific polymorphisms in the purified genomic DNA samples. The genotyping test included specific sequences for forward and reverse primers, as well as two TaqMan^®^ minor groove binder (MGB) probes with non-fluorescent quenchers (NFQ). One probe was VIC^TM^-labeled to detect the wild-type sequence, and the other was FAM^TM^-labeled for detection of the mutated gene sequence. The genotyping analysis was performed on a Real-Time PCR 7500 Fast system (Applied Biosystems, USA).

### Ethical statement

All participants provided informed consent, and the study was approved by the Ethics Committee of the University Clinical Hospital Mostar (approval number 500/19).

### Statistical analysis

The statistical analysis was performed in IBM SPSS Statistics software, version 25 (Armonk, NY: IBM Corp.). The Kolmogorov–Smirnov test was used to determine whether the data were normal. The mean and standard deviation (SD) or median and interquartile range (IQR) were used to express numerical variables. The Spearman’s correlation coefficient was used to examine the relationships between the variables. Descriptive statistics were used to summarize the characteristics of the study population, including median, standard deviation, and IQR. The frequency of *MTHFR* gene mutations (C677T and A1298C) was calculated, and the distribution of genotypes was presented as percentages. To determine the significance of differences in homocysteine values according to the *MTHFR* gene polymorphisms, a Kruskal–Wallis test was performed. Additionally, correlations between homocysteine levels and vitamin B12, as well as folic acid, were assessed using Spearman’s Rho correlation coefficient. The significance level was set at *P* < 0.05.

## Results

The study included a total of 102 pregnant women with ages ranging from 19 to 43 years. Table 1 presents the basic characteristics of the study population, including age, BMI, and serum levels of active B12, folic acid, and homocysteine. Among the participants, 33 (32.4%) pregnant women had homocysteine levels below the reference value. It was found that 8.8% of pregnant women had low folic acid values (below the reference value), while 2% had high values (above the reference value).

**Table 1:** Characteristics of pregnant women (*n* ═ 102)

	**Mean**	**SD**	**Median**	**IQR**
Age (years)			30.0	28.0 – 31.2
BMI (kg/m^2^)	23.75	3.78		
Active B12 (pmol/L)			49.7	34.8 – 61.8
Folic acid (nmol/L)			14.2	9.6 – 24.1
Homocysteine (µmol/L)			5.0	4.3 – 5.9

SD: Standard deviation; BMI: Body mass index; IQR: Interquartile range.

Table 2 provides the frequency distribution of *MTHFR* gene mutations in the study population (*n* ═ 102). Among the participants, 56.9% had the *MTHFR* C677T gene mutation, with 43.1% being wild type (CC), 47.1% heterozygous (CT), and 9.8% homozygous (TT). Regarding the *MTHFR* A1298C gene mutation, 89.2% of the pregnant women carried this mutation, with 10.8% being wild type (AA), 40.2% heterozygous (AC), and 49.0% homozygous (CC).

**Table 2:** Frequency of *MTHFR* mutations in the study population (*n* ═ 102)

	**Number (%)**
*MTHFR* C677T	
Wild type (CC)	44 (43.1)
Heterozygote (CT)	48 (47.1)
Homozygote (TT)	10 (9.8)
*MTHFR* A1298C	
Wild type (AA)	11 (10.8)
Heterozygote (AC)	41 (40.2)
Homozygote (CC)	50 (49.0)

### *MTHFR* C677T gene

To determine the significance of differences in homocysteine values according to the *MTHFR* gene polymorphisms, a Kruskal–Wallis test was performed. This analysis revealed that there were no significant differences in homocysteine values among individuals with different *MTHFR* C677T and A1298C genotypes (Tables 3 and 5).

**Table 3:** Homocysteine values according to the *MTHFR* C677T polymorphism

	**Median**	**IQR**	P*
Wild type (CC)	5.57	4.23 – 6.32	0.245
Heterozygote (CT)	4.93	4.29 – 5.69	
Homozygote (TT)	5.36	4.35 – 7.15	

*Kruskal–Wallis test; IQR: Interquartile range.

For the wild type of the *MTHFR* A1298C gene, homocysteine was significantly correlated with vitamin B12, as well as folic acid (Table 4). Pregnant women with higher serum levels of folic acid and vitamin B12 had lower levels of homocysteine.

**Table 4:** Correlation of B12 vitamin and folic acid serum levels with homocysteine according to genetic polymorphism of the *MTHFR* C677T gene

	**Active B12 (pmol/L)**		**Folic acid (nmol/L)**	
	**Spearman’s Rho**	***P***	**Spearman’s Rho**	***P***
Wild type (CC)	−0.449**	0.002	−0.497*	0.001
Heterozygote (CT)	−0.172	0.244	−0.257	0.078
Homozygote (TT)	−0.164	0.651	−0.564	0.090

*Correlation is significant at the 0.05 level (2-tailed). **Correlation is significant at the 0.01 level (2-tailed).

### *MTHFR* A1298C gene

There were no significant differences in homocysteine values according to the *MTHFR* C677T polymorphism (Table 5).

Tables 4 and 6 show the correlation between homocysteine levels and vitamin B12, as well as folic acid, according to the genetic polymorphisms of *MTHFR* C677T and A1298C, respectively. Among individuals with the wild type of *MTHFR* A1298C gene, there was a significant negative correlation between homocysteine levels and vitamin B12 (Spearman’s Rho ═ −0.282, *P* ═ 0.401) and folic acid (Spearman’s Rho ═ −0.355, *P* ═ 0.285). Similarly, in heterozygotes for the *MTHFR* A1298C gene, significant negative correlations were observed between homocysteine levels and vitamin B12 (Spearman’s Rho ═ −0.477, *P* ═ 0.002) and folic acid (Spearman’s Rho ═ −0.547, *P*< 0.001). These correlations indicate that higher serum levels of vitamin B12 and folic acid are associated with lower homocysteine levels in these specific genotypes.

**Table 5:** Homocysteine values according to the *MTHFR* A1298C polymorphism

	**Median**	**IQR**	P*
Wild type (AA)	4.52	3.95 – 5.72	0.073
Heterozygote (AC)	5.30	4.95 – 6.20	
Homozygote (CC)	4.66	4.01 – 5.92	

*Kruskal–Wallis test; IQR: Interquartile range.

**Table 6:** Correlation of B12 vitamin and folic acid serum levels with homocysteine according to genetic polymorphism of the *MTHFR* A1298C gene

	**Active B12 (pmol/L)**		**Folic acid (nmol/L)**	
	**Spearman’s Rho**	***P***	**Spearman’s Rho**	***P***
Wild type (AA)	−0.282	0.401	−0.355	0.285
Heterozygote (AC)	−0.477**	0.002	−0.547**	<0.001
Homozygote (CC)	−0.157	0.276	−0.221	0.122

**Correlation is significant at the 0.01 level (2-tailed).

There were no significant differences in homocysteine values based on *MTHFR* C677T and A1298C genotypes. These findings contribute to the existing knowledge, providing insights into the relationship between *MTHFR* mutations and homocysteine levels in pregnant women. We tested two genetic variants, *MTHFR* C677T and *MTHFR* A1298C, for compliance with the Hardy–Weinberg law. The *P* value for the *MTHFR* C677T variant was 0.76141, and the *P* value for the *MTHFR* A1298C variant was 0.96402, suggesting that both observed genotype frequencies in our population are in agreement with the expected frequencies under Hardy–Weinberg equilibrium. This finding supports the notion that there are no significant deviations from the principles of genetic inheritance for these two *MTHFR* variants in our study population.

## Discussion

The aim of this research was to investigate the frequency of two clinically significant *MTHFR* gene mutations and the vitamin status of pregnant women at the University Clinical Hospital in Mostar. We explored the association of homocysteine levels with the *MTHFR* polymorphism, vitamin B12 values, and total folate. Out of 102 pregnant women in our study, 56.9% had the C677T polymorphism, mostly in the heterozygous form, while 89.2% had the A1298C polymorphism, predominantly in the homozygous form. The prevalence of *MTHFR* gene mutations varies across different populations, with higher rates observed in certain ethnic groups. The C677T polymorphism is more prevalent among Caucasians in the USA (20%–40%), while its occurrence is lower in blacks (up to 2%). The A1298C polymorphism is found in 7%–12% of individuals in North America, Australia, and Europe, with lower rates observed among Spaniards (4%–5%), Chinese (1%–4%), and Asians (1%–4%) [17]. In Bosnia and Herzegovina, the frequency of the C677T allele was found to be 37.5%, higher than that reported in several other European populations [18]. Our study revealed an unusually high prevalence (89.2%) of the A1298C polymorphism in our population, a novel finding compared to the existing literature.

Concerning vitamin status, we identified that 7.8% of pregnant women had total folate levels below the reference values. According to Wartanowicz et al., plasma folate levels ≥14.9 nmol/L are considered the optimal cut-off value, associated with a very low risk for neural tube defects [18]. In our study, we found that the median level of total folate was 14.2 nmol/L, which falls slightly below the optimal cut-off value of ≥14.9 nmol/L associated with a very low risk for neural tube defect. This indicates a significant proportion of women with suboptimal folic acid levels, highlighting the potential for improvement in folate supplementation strategies. Adequate folate intake has been shown to significantly reduce the risk of neural tube defects, making it a critical consideration during pregnancy.

We also assessed the level of active vitamin B12, holotranscobalamin, which is considered a superior indicator of vitamin B12 status compared to total vitamin B12. However, reports on the frequency of vitamin B12 deficiency have been inconsistent in the literature [19]. The use of different markers and threshold values for diagnosis makes interpretation and comparison of results challenging. Moreover, the lack of consensus on a reference value for vitamin B12 during pregnancy remains a significant challenge [18]. Some researchers, such as Köbe et al. proposed cut-off points of < 186 and < 180 pmol/L for total B-12, and < 62.2 and < 67.5 pmol/L for holotranscobalamin in the first and second trimesters, respectively, to indicate an increased likelihood of impaired intracellular B-12 status, with no significant variation observed across different ethnic groups [19, 20]. Our study revealed that 83.3% of pregnant women had vitamin subclinical B12 deficiency based on these criteria.

As for homocysteine, values were within the normal range for all participants, with a median plasma homocysteine level of 5 µmol/L, consistent with other reports [21]. These results provide valuable epidemiological data that can aid in planning pregnancy management and interventions focused on nutrition and supplements to enhance perinatal outcomes.

Our investigation did not find a significant correlation between *MTHFR* gene mutations and homocysteine levels, which contrasts with some previous reports. Studies on general populations and pregnant women have shown associations between *MTHFR* polymorphisms and elevated homocysteine levels [22–24]. The lack of association in our study may be attributed to the vitamin supplements containing 5-MTHF, a methyl group donor, which potentially bypasses the weakened enzymatic conversion caused by the mutation. Furthermore, it has been observed that high-level folate intake can mitigate the adverse pregnancy outcomes associated with hyperhomocysteinemia induced by *MTHFR* variant genotypes [25]. However, we did find a statistically significant correlation between homocysteine levels and vitamin B12 and total folate in individuals with the wild-type of the *MTHFR* A1298C gene and heterozygous individuals for the *MTHFR* A1298C polymorphism. The reason for this correlation remains unclear. We hypothesized that in conditions of weakened function of MTHFR, there would be an association between homocysteine levels and vitamin B12 and folic acid, essential dietary components. Without folic acid as a substrate and vitamin B12 as a cofactor in the methylation cycle, homocysteine accumulates. Independently of *MTHFR* mutations, we observed a negative correlation between homocysteine levels and vitamin B12 (Spearman’s Rho ═ −0.290, *P* ═ 0.003) as well as between homocysteine levels and folic acid (Spearman’s Rho ═ −0.400, *P*< 0.001). These findings align with those reported by other authors [26, 27]. Barbosa et al. [28] studied gene-nutrition interactions between *MTHFR* polymorphisms and vitamin status and found that elevated homocysteine levels were associated with low serum folate status, independently of gene polymorphisms.

We acknowledge that a limitation of our research is the relatively small number of samples included in the study and the lack of epidemiological data. A larger sample size would provide more robust and representative results. Despite this limitation, our study offers valuable insights into the association between *MTHFR* gene polymorphisms, vitamin status, and homocysteine levels in pregnant women during folic acid supplementation. Longitudinal studies are needed to investigate the effects of *MTHFR* gene variants and homocysteine levels throughout pregnancy. Additionally, exploring the impact of dietary and lifestyle factors on homocysteine metabolism and assessing potential interventions, such as folic acid and vitamin B12 supplementation, are essential for improving maternal and fetal outcomes. Moreover, investigating the relationship between *MTHFR* gene polymorphisms, homocysteine levels, and pregnancy complications, as well as placental inflammation and perinatal outcomes, will provide a comprehensive understanding of the genetic and environmental influences on maternal health.

## Conclusion

Our cross-sectional study found a high prevalence of *MTHFR* gene mutations, particularly A1298C, among pregnant women in the Herzegovinian population. We observed negative correlations between homocysteine levels and both vitamin B12 and folic acid. No correlation was found between the mother’s genotype and serum values of homocysteine. A large proportion of pregnant women had suboptimal folate status and a subclinical vitamin B12 deficiency. Our findings emphasize the benefits of mandatory folic acid supplementation and targeted interventions to enhance maternal nutrition and minimize adverse pregnancy outcomes. Adequate dietary intake, including mandatory supplementation with 5-methylene tetrahydrofolate, could have a crucial impact on total folate levels. Additionally, the significant correlation between homocysteine levels and vitamin B12 and folic acid underscores the importance of ensuring sufficient levels of these vitamins to prevent adverse pregnancy outcomes.
